# CADe System Integrated within the Electronic Health Record

**DOI:** 10.1155/2013/219407

**Published:** 2013-09-17

**Authors:** Noelia Vállez, Gloria Bueno, Óscar Déniz, María del Milagro Fernández, Carlos Pastor, Miguel Ángel Rienda, Pablo Esteve, María Arias

**Affiliations:** ^1^VISILAB Group, E.T.S.I. Industriales, University of Castilla-La Mancha, 13071 Ciudad Real, Spain; ^2^Department of Radiology, University General Hospital of Ciudad Real, 13005 Ciudad Real, Spain; ^3^Department of Radiology, Hospital of Alcázar de San Juan, 13600 Alcázar de San Juan, Spain

## Abstract

The latest technological advances and information support systems for clinics and hospitals produce a wide range of possibilities in the storage and retrieval of an ever-growing amount of clinical information as well as in detection and diagnosis. In this work, an Electronic Health Record (EHR) combined with a Computer Aided Detection (CADe) system for breast cancer diagnosis has been implemented. Our objective is to provide to radiologists a comprehensive working environment that facilitates the integration, the image visualization, and the use of aided tools within the EHR. For this reason, a development methodology based on hardware and software system features in addition to system requirements must be present during the whole development process. This will lead to a complete environment for displaying, editing, and reporting results not only for the patient information but also for their medical images in standardised formats such as DICOM and DICOM-SR. As a result, we obtain a CADe system which helps in detecting breast cancer using mammograms and is completely integrated into an EHR.

## 1. Introduction

Mammograms are difficult to interpret. This fact is especially aggravated in screening campaigns. In these campaigns, radiologists have to examine a large number of mammograms of asymptomatic patients to try to detect breast cancer in its early stages. Therefore, mammograms play a very important role in early detection of this type of cancer. The sensitivity of mammographic studies depends on the quality of the images and the radiologist experience and concentration levels. CADe (Computer-Aided Detection) and CADx (Computer Aided Diagnosis) systems can help radiologists and increase the detection and diagnostic precision by offering a second opinion. This second opinion has lower price than that of a human radiologist. These systems detect suspicious forms in mammograms and mark and classify them according to their malign or benign character. These systems also provide visual results for radiologists [1].

An Electronic Health Record (EHR) is a collection of relevant medical data about the life of patients stored in electronic format [2, 3]. It compounds specific information about patients such as allergies, prescribed medications, immunizations, or major diseases in addition to other information from tests that were performed and the results obtained in them. This information can provide information feedback to health care professionals [4]. Therefore, in an EHR it is possible to find several types of data with different formats, such as, image, video, audio, or text.

Given the possibility that each institution develops its own EHR using different file formats, some standards to facilitate information exchange have been published. The main standard in medicine to store images and results is the Digital Imaging and Communication in Medicine (DICOM) standard [5].

The integration of a display system within the EHR facilitates accessing to patients textual information and displaying medical test images in a complete working environment. This makes all the information concerning a patient accessible from anywhere with appropriate access restrictions.

Most of the current CADe systems are independent of the other applications installed in clinics and hospitals. There is a need to integrate them [6, 7]. Using information from both the EHR and the CADe system helps to provide a more complete diagnostic [8].

This paper shows the integration of a CADe system within the EHR. Thus, a comprehensive working environment to radiologists which facilitates the integration together with the visualization and aided tools within the EHR has been developed. The aim of the CADe system is to be a tool for displaying medical images and helping in breast cancer detection. Moreover, an EHR in which a CADe system is integrated to complete its functionality has been developed.

Sections 2 and 3 present a study of the current EHRs and CADe systems for mammography in addition to their advantages and disadvantages.  Section 4 presents a complete model of the CADe system and the EHR developed. This model is based on the use of a software development methodology, the study of the physical architecture of the system, and compatibility requirements with the most used image format in medicine. Finally, the results and conclusions are outlined in Sections 5 and 6.

## 2. Electronic Health Record

Nowadays, many health centres offer or are being adapted to offer the possibility of accessing medical information using information systems and advanced technology. Hospitals in Middle East countries [9, 10], most United States clinics [11], and health centres of European countries [12] are some examples.

The use of EHRs has the following advantages [13].

1. Availability and accessibility of data at any time and anywhere.
2. Effective patient treatment enhancement by the ability to use saved data for further studies.
3. Quick and easy access to data.
4. Reduction of the number of unnecessary tests. Specialists can easily access previous test results that may be still valid.
5. Reduction in information loss.
6. Reduction of costs. Using EHRs it is not necessary to print results.
7. Reduction of data redundancies.
8. Data access security enhancement.

In contrast, the potential risks that should be considered for the successful design and implementation of EHRs are [14, 15].

1. Tedious data entry.
2. Unfriendly user interfaces.
3. Creating additional and unusual work for physicians.
4. Interruptions or alterations in the usual workflow.
5. Lack of network connectivity.
6. Update failures.
7. Using nonstandardized terms.
8. Security failures.
9. Rejection by system users.
10. Large response times.
11. Programming errors.
12. Limited documentation.

Although there are several studies that have implemented an EHR, we focus our attention on integration of CAD systems within the EHR. As it stands today, there is no one step by step process for developing EHRs. While some authors use Web Services (WS) to access to databases (DB) [2], others use XML files for storing patient data or processing stored data obtained with medical images [16–18].

Other authors use a Web Service Oriented Architecture [19], SOA, that provides interoperability between different systems and programming languages, usability to deploy Web applications, and possibility of reusing implemented functions. SOAs also facilitate application development. In contrast, in some cases, the use of SOA may be a problem because they use a protocol based on plain text in which requests can be larger than in other protocols. Therefore, they can increase the elapsed time and pending client connections.

The EHRs that use XML files to store all the patient information suffer from a large data dispersion along several files. This data dispersion may cause data loss or duplication. Thus, health care record fragmentation must be reduced [20]. Retrieving information about various patients or compiling statistics with a DB offers greater simplicity and speed than XML files.

## 3. Computer Aided Detection Systems

The computer-aided diagnosis process can be defined as a diagnosis given by radiologists who use an automatic image analysis system to assist them [21]. The system's output acts as a second reader opinion. This second opinion helps radiologists to find previously missed important regions.

The ability to detect lesions by examining a radiological image varies depending on the circumstances of the observer. Fatigue, distraction, or stress are some examples in which a radiologist can ignore early cancer signs. Therefore, a radiologist could detect lesions in some cases and not in others. These facts make CAD systems more suitable for sessions in which a radiologist examines a large number of images with low rate of lesion occurrence.

Most work on CAD systems focuses on breast cancer. The interpretation of mammograms is not easy; even expert radiologists miss early signs of cancer in between 10% and 30% of cases. The use of CAD as a second reading increased cancer detection by 15% [21]. The purpose of computer-aided diagnosis is to provide a second opinion for reducing the number of false negatives (FNs) and false positives (FPs). However, radiologist do not trust CAD results since sometimes CADs can provide a large number of FPs even when the diagnosis was correct [22].

The use of digital image formats to store mammograms, either by direct acquisition of these images or by digitization of printed mammograms, facilitates the work of radiologists in diagnosing breast cancer. There are two types of CAD systems according to the form by which digital images are obtained: based on digitized screen-film mammograms (SFMs) and based on full-field digital mammograms (FFDMs). SFMs CAD systems have one disadvantage: there is noise in SFMs images produced during the scanning process. By contrast, the use of FFDMs CAD systems offers less noise, larger colour range, and higher contrast than the previous ones [23]. All commercial CAD systems that have obtained FDA approval use FFDMs as input. In general, both types of CAD systems obtain similar accuracy.

In order to make diagnostic decisions, radiologists look for a number of lesion characteristics: contour, density, location, size, and so forth. These characteristics are defined by the ACR (American College of Radiology) [24].

In recent years, many researches have dedicated time, personal and economical efforts to develop this kind of application [25]. In particular, most of the work has been dedicated to the development of the detection and classification functions of CAD systems [26]. Currently, there are at least three mammography CAD systems approved by the FDA: *R2 Technology, Inc.* [27], *Intelligent Systems Software, Inc.* [28], and *CADx Medical Systems.* [29].

However, most of these systems are independent of the other applications that are installed in clinics and hospitals. It is common to find other applications that access images stored in digital format or provide mechanisms to digitize mammographic films in medical centres [6].

## 4. Materials and Methods

Medical images in DICOM format and test results files in DICOM-SR format (DICOM structured report) are used as input and output files, respectively, by the proposed CAD system. Moreover, it is possible to view images stored in other file formats on the application like *jpg*, *png*, *pgm*, and so on.

In order to design and build both applications, the CAD system for mammography and the EHR, several paradigms have been proposed in the literature. Bernstein et al. [30] describe four different methodologies for developing an EHR: using a semantic model, using the generic model of three layers, using a model based on middleware, and through a model based on communication.

The paradigm proposed in this paper is based on a combination of the generic three-layer model with client-server architecture and a software development methodology such as Unified Process (UP) [31]. DICOM-SR format has been used to store CAD results.

### 4.1. Images and Reports

Systems based on output files that work in medical institutions do not always satisfy the requirements of some standards that specify the encoding, the storage, and the transfer protocols of medical data. These nonstandardized systems hinder the information interchange between different hospitals.

The DICOM standard was developed to make possible the communication between different clinical and hospital information systems and the appropriate medical image storage and retrieval. In 1993, its creators, ACR (American College of Radiology) and NEMA (National Electrical Manufacturers Association), presented the third version of the standard [5]. The standard describes the file format, the information fields that appear in it, and the image or the images contained in a DICOM file to provide a means to access and interpret all the information of different medical systems. Following the DICOM standard, images are stored together with patient, doctor, or clinic data and the information about results or measures. Therefore, the display system must be able to handle this type of image files.

In addition, DICOM-SR is a part of the DICOM standard that refers to the storage and transmission of clinical reports. The data is stored in a file format similar to the image one.

Another problem is that in some storage systems, results are based on the use of pure text. The pure text is difficult to translate into other languages, may contain ambiguities, and searches using it are not efficient.

Using DICOM-SR format files to store and display the reports in a structured way is a solution to the above problems and adds several advantages [32].

1. The SR specifies rules that define how structured documents that contain medical results should be created, stored, and transmitted.
2. Such documents may contain references to other DICOM files such as images and audio files.
3. SR uses a standard terminology to avoid the ambiguity of natural language, facilitate the automatic interpretation of the content, and improve search.

**Figure 1:**
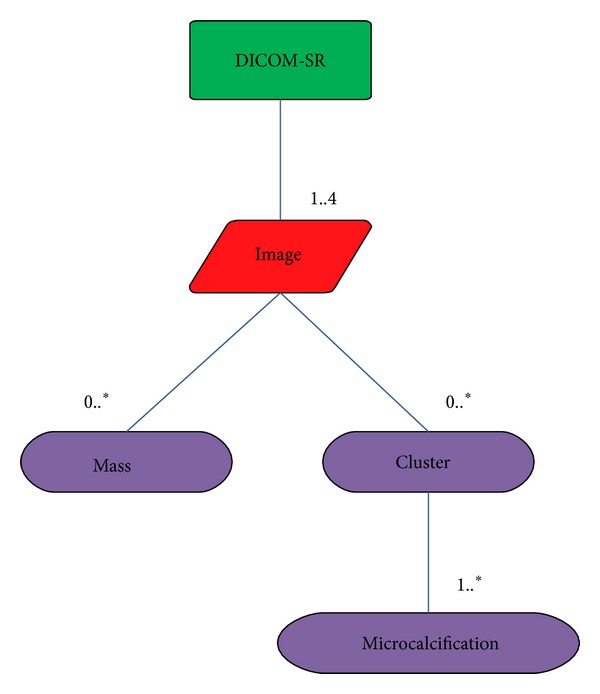
Mammographic structured report hierarchy.

In our case, it is necessary to store information about images and lesions found in mammograms. The main lesions in breast images are masses and microcalcifications [33].  Figure 1 shows the hierarchy data results that must be saved in case of working with a regular mammographic study which contains four images (a craniocaudal projection (CC) and a mediolateral oblique (MLO) projection by each breast).

### 4.2. Application Development

For developing the complete application we focus our attention, first, on the physical structure. Currently, the ability to access applications from anywhere and any time is desirable. There is an increasing necessity to use physical architectures that facilitate data access from different locations and devices. To deal with this situation, the client-server architecture is a great solution. In this architecture, each system that operates as a client creates an information demand to the server system. The server systems provide the information required responding to the demand made by the client.

Applications based on this type of hardware architecture do not need to change the whole system with every update. It is sufficient to make small changes in the client side or in the server side independently. It is also possible to move, change, or update some computers on the network without any repercussions on the rest of devices. Sometimes it is possible that changes in server applications affect, minimally, client applications or vice versa.

The EHR proposed in this paper bases its operation on the client-server architecture. This is an application that can be installed on an application server and can be accessed through a web browser from any computer with network and user privilege access. The application server is responsible for carrying out the appropriate operations and facilitating the communication with the DB to provide the necessary information to the clients. The Web browser is not usually connected directly to the application server. In most cases, the connection is done through a web server that acts as an intermediary. The web server, the application server, and the data server can be found in the same physical machine or separated in different computers.

Traditionally, this type of applications has been developed using Common Gateway Interface (CGI). A few years ago Java Servlets appeared for programming server side applications [34]. Servlets are small programs that run on the server after a request made by the client. Java Server Pages (JSP) is an improvement of Servlets and facilitates the work making programming easier and reducing the time used for this task. For coding on the server side Microsoft created Active Server Pages (ASP.NET). We decided to use ASP.NET for developing the EHR as in [35].

**Figure 2:**
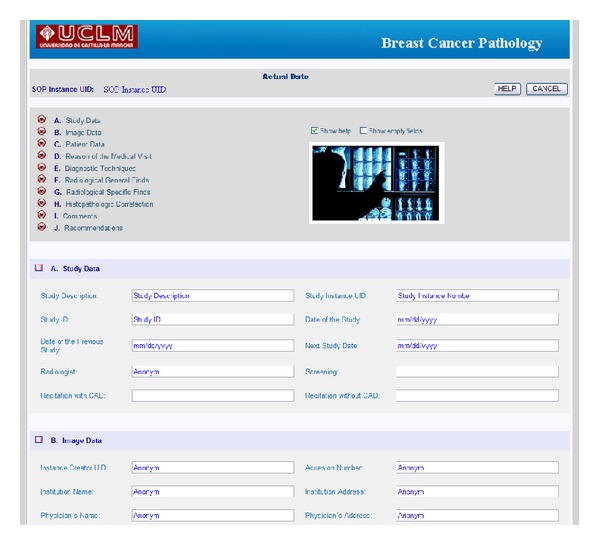
Extract of the implemented EHR principal view.

**Figure 3:**
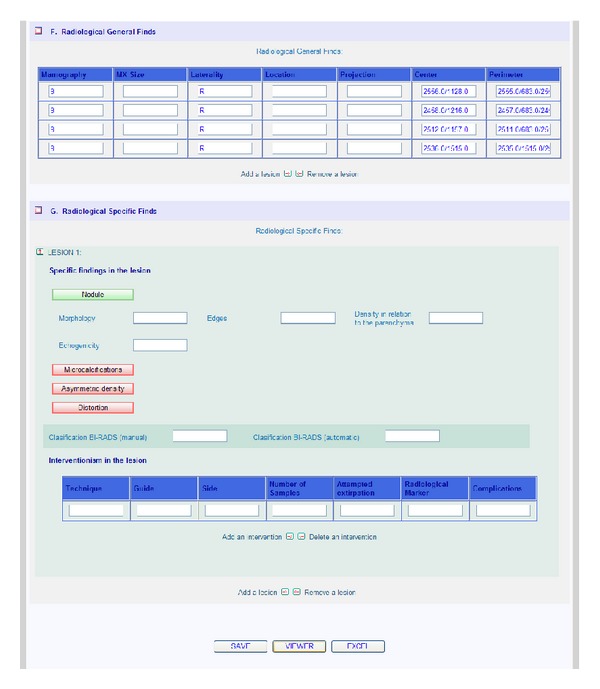
Extract of the implemented EHR information about mammographic findings and CAD link with the button “VIEWER.”

The EHR has been developed based on radiologic templates and the ACR standard [24]. These templates are created from the requirements obtained in different meetings and questionnaires with some specialists like in [36]. Moreover, it is important to take into account some EHR specific requirements [37]. When the user starts the application, the EHR shows only the basic fields. If the user needs other data or information, it is possible to display more fields by pressing the corresponding buttons. This makes managing patient information easier. Figures 2 and 3 show the appearance of the mammographic EHR.

In order to develop and integrate the CAD system within the EHR, web oriented programming languages should be used. Java and JavaScript are examples of this type of web programming languages. Specifically, Java Applets are used to develop the CAD system. The use of Java Applets offers the following advantages.

1. Applets can run on any operating system which has previously installed a JVM (Java Virtual Machine).
2. New versions of the JVM are able to run applets created for previous versions.
3. Applets are supported by most Web browsers.
4. You can have full access to the machine on which the applet is running if the user permits it.
5. The work can be moved from servers to clients. Sometimes, all the work of several clients should be centralized on the server to prevent overloading and reduce queues.

Although Java Applets have some security restrictions, such as the impossibility to access client resources, it is possible to deal with them packaging the Applet in a JAR-file, that enables to bundle multiple files into a single archive file and sign it. Signed applets do not have the security restrictions that are imposed on unsigned applets and can run outside the security sandbox. Users who verify the signature can grant the JAR-bundled software security privileges.

The EHR and the CAD system have a bidirectional communication. The EHR is responsible for providing the necessary parameters for the CAD initialization by using the 〈applet〉 label existing in HTML or the 〈object〉 label in HTML5. Once the CAD is initialized, the communication between the EHR and the CAD is made by JavaScript. The JSObject class allows Java and Java Applets to manipulate objects that are defined in JavaScript. The EHR also has two combo-boxes to select the images that are loaded in the CAD panels. If a different value is selected in any of the combo-boxes, a call to a JavaScript function is made. This function sends the new parameters to a function of the CAD system that handles the change of the images loaded. Moreover, the CAD system is able to communicate results to the EHR by means of calling JavaScript functions residing in the EHR. After receiving the results, the EHR is the responsible for their storing, retrieval, and use.

SR files are used to store CAD results. Once a radiologist select, the final results of the CAD system it is possible to store them in a SR file and in the EHR database. In this step, it is important to control that there are no inconsistencies between data stored in SR files and data stored in the EHR database. For creating SR files the *dcm4che* library is used [33]. To store the results in the EHR database, the CAD system calls to a JavaScript function of the EHR.

For the code structure and the software development process of the application we propose to use the three-layer architecture and the Unified Process (UP). The three-layer model performs a code division according to the responsibilities that has every part of the application code. The classical division of the code divides it into a presentation layer, a domain layer, and a data layer. In our application the use of this architecture

1. facilitates reuse and migration,
2. improves human resources distribution,
3. makes maintenance easier,
4. limits application changes propagation between layers.

Finally, the software development process is selected according to the software structure that has to be developed. The developed software quality often depends on the methodology used for its construction. Currently, one of the more used methods to develop a complete system is the UP [31]. This development methodology is perfectly compatible with the three-layer architecture. The design is made from the use cases and the system architecture of the application. It is iterative, incremental and tries to predict the risks from the start of the project. The main reasons why we select this development methodology are as follows.

**Figure 4:**
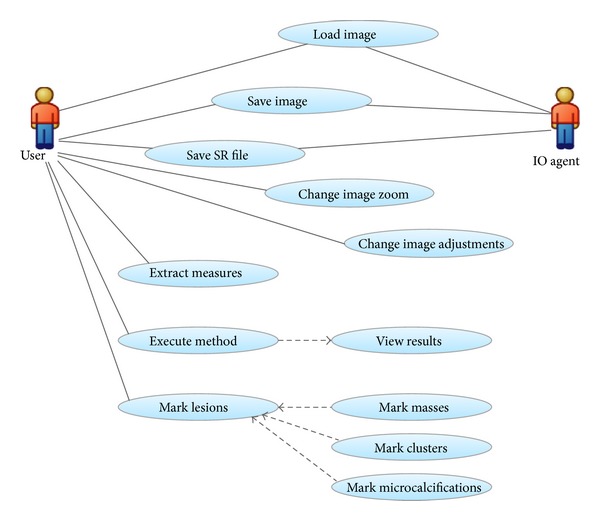
CAD application use case diagram.

1. As the system is developed according to the use case diagrams, in which each use case specifies the system functionality, it contains all the functions that should be fulfilled (Figure 4).
2. The use of UP minimizes the effects of risks by identifying them from the start.

### 4.3. Image Preprocessing

The idea of using computers in radiographic image analysis is not new. In 1964 an automatic system that determines the proportion of the heart in the thoracic chest X-ray was proposed [38]. In 1967 a system for automatic analysis of mammograms based on bilateral comparison was developed [39]. In 1975 an algorithm for detection of microcalcifications in mammograms based on identifying peaks of grey values was described [21]. After these developments, and due to poor image quality, there was no significant progress in this issue until the late 80s.

Over recent years, various techniques have been developed to detect and automatically characterize masses, microcalcifications, asymmetries, and other lesions in mammography. In addition, many studies on the development of CAD systems have been made [26]. In recent decades there have been important advances in signal processing. The rapidly increasing processing power is responsible for these advances. This increment represents the viability of the use and the study of new algorithms with higher computational costs than before.

**Figure 5:**
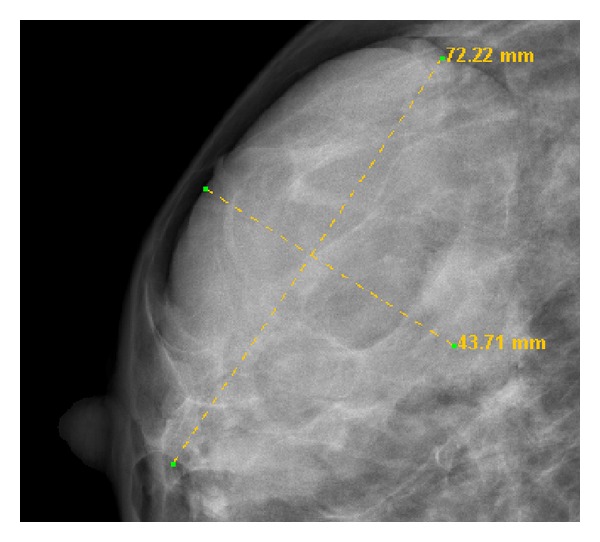
Lesion measures. The values represent the real distance in millimetres.

Although it is very important to detect and classify masses and microcalcifications, the correct image preparation using preprocessing and editing tools is a crucial step [40, 41]. When digital images and a display system are used, the system must be able to simulate the actual tools that are used by specialists in printed images analysis (Figure 5). Changing the size of the image that is being displayed or performing actions on it such as contrast enhancement or brightness adjustment helps to perform tasks faster, easier, and in a less costly way than if they were carried out manually.

**Figure 6:**
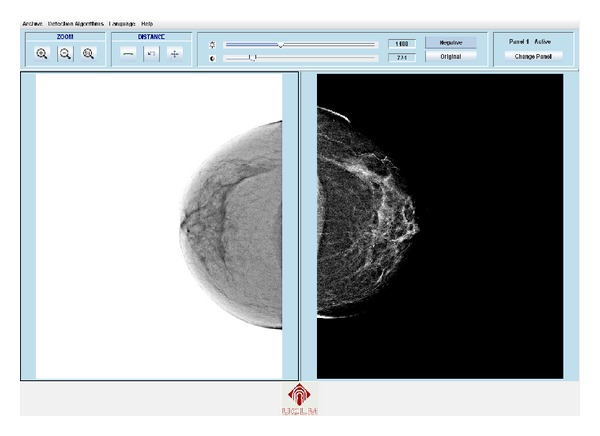
Negative, brightness, and contrast effects on images.

There are several preprocessing techniques that improve image perception highlighting some areas, illuminating the image, or removing the noise [40, 41]. Contrast enhancement is one of the most widely used techniques for improving image visualization and has great application oriented to medical images. Improvements that are mainly achieved are intermediate zones of detail enhancement, edge definition, and overall image contrast [42]. In this case, options for image preprocessing such as changing the brightness, the contrast, and obtaining the negative were added to the visualization part of the CAD application (Figure 6). In some cases it is easier to identify lesions in the image obtained by inverting the original colour.

### 4.4. Image Processing

In addition to the preprocessing tools the CAD system should have tools for the detection of two main breast lesions: masses and microcalcifications.

An automatic filter to highlight the visibility of microcalcifications on breast tissue is usually applied. Sometimes it is sufficient to detect clusters of these lesions and not each microcalcification individually [21].

A common practice in mass detection is the bilateral comparison of asymmetric densities. The first stage is characterized by the correct alignment between left and right mammograms. Once the images are well aligned, a subtraction between grey values of the two images is performed, obtaining in this step the density difference [21]. However, this technique has problems because there are natural density differences between the two breasts and this could cause a significant number of false positives.

When processing mammograms it is possible to take two different approaches: make algorithms for contrast enhancement that allow distinguishing lesions from background or performing algorithms to detect and mark them. In [33] several algorithms for helping in detecting masses, microcalcifications, and distortions were developed. These algorithms were integrated into the CAD tool.

(i) *Fuzzy Logic Clustering*. Fuzzy logic clustering helps in mass contour outline. Fuzzy sets are a generalization of conventional set theory introduced by Zadeh in 1965 as a mathematical way to represent uncertainties [43]. Fuzzy set theory applied to image segmentation is a fuzzy partition of the image data, *I*(*x*, *y*) = {**x**_1_, **x**_2_,…, **x**_**n**_}, into *c* fuzzy subsets or *c* specified classes. That is, {**x**_**m**_(*i*, *j*):→[0,1] : *m* = 1,…, *c*}, which replaces a crisp membership function that divides the image into *c* regions by means of a nonfuzzy segmentation process. Thus, image data is clustered into the *c* different classes by using an unsupervised fuzzy C-mean (FCM) clustering algorithm. This is achieved by computing a measure of membership, called fuzzy membership, at each pixel [44]. The fuzzy membership function, constrained to be between 0 and 1, reflects the degree of similarity between the data value at that location and the centroid of its class. Thus, a high membership value near 1 means that the data value (pixel intensity) at that location is close to the centroid for that particular class. The FCM algorithm is then formulated as the minimization of the squared error with respect to the membership functions, *U*, and the set of centroids, {*V*} = {*v*_1_, *v*_2_,…, *v*_*n*_}:

(1) $$\begin{array}{c} J\left(U,V:X\right)=\sum_{i=1}^{c}\;‍\sum_{k=1}^{n}{\left(u_{ik}\right)}^{m}||x_{k}-v_{i}||, \end{array}$$

where *u*_*ik*_ = *u*_*i*_(**x**_**k**_) is the membership of **x**_**k**_ in class *i* and *m* ≥ 1 is a weighting exponent of each fuzzy membership.

The FCM objective function, (1), is minimized when high membership values are assigned to pixels whose intensities are close to the centroid for its particular class and low membership values are assigned when the pixel intensity is far from the centroid. Taking the first derivatives of (1), with respect to *u*_*ik*_ and *v*_*k*_, and setting those equations to zero yield necessary conditions for (1) to be minimized. Then *u*_*ik*_ and *v*_*i*_ are defined as follows:

(2) $$\begin{array}{c} u_{ik}={\left[\sum_{j=1}^{c}{\left(\frac{||x_{k}-v_{i}||}{||x_{k}-v_{j}||}\right)}^{(2/\left(m-1)\right)}\right]}^{-1},\\ v_{i}=\frac{\sum_{k=1}^{n}{\left(u_{ik}\right)}^{m}x_{k}}{\sum_{k=1}^{n}{\left(u_{ik}\right)}^{m}}\;\forall i,k. \end{array}$$

Iterating through these conditions leads to a grouped coordinate descent scheme for minimizing the objective function. The stop criterion is determined for each iteration as *E*_*i*_ < *ε* where

(3) $$\begin{array}{c} E_{i}=\sum_{i=1}^{c}||v_{i,t+1}-v_{i,t}||\;\forall t. \end{array}$$

The resulting fuzzy segmentation can be converted to a hard or crisp segmentation by assigning each pixel solely to the class that has the highest membership value for that pixel. This is known as a *maximum membership* segmentation. Once the method has converged, a matrix *U* with the membership or degree to which every pixel is similar to all of the *c* different classes is obtained. Every pixel is assigned the class for which the maximum membership is found. That is, if max⁡(*u*_*ik*_) = *u*_*ik*_, then **x**_**k**_ is assigned the label associated with class *i*. Further modifications of the FCM algorithm may be done by introducing more terms to the objective function (1), for example, in order to cope with noisy images [44].

The result of the FCM algorithm may be rather variable according to the number of selected clusters, *c*, and the position of the centroids, {*V*}. In order to apply the FCM algorithm to the problem of interest a proper configuration of both *c* and {*V*} has been found from analysis of image histograms [45].

(ii) *Contrast Enhancement Using β-Spline*. It tries to get a picture with the effect of raised areas from the original image using the 3rd order *β*-Spline transform with an expansion degree of 1:

(4) $$\begin{array}{c} B_{1}^{3}{\left(z\right)}^{-1}=\frac{6}{z+4+z^{-1}}. \end{array}$$

The *β*-spline filter, (*B*_1_^3^)^−1^, has been implemented recursively by using a causal (*f*_*c*_(*z*)) and anticausal (*f*_ac_(*z*)) filter:

(5) $$\begin{array}{c} f_{c}\left(z\right)=\frac{6}{1-z_{1}z^{-1}}\\ f_{\text{ac}}\left(z\right)=\frac{-z_{1}}{1-z_{1}z} \end{array}$$

with *z*_1_ = −0.268.

Rewriting the above equation with *f*_*c*_ and *f*_ac_ filters, the equations will be as follows:

(6) B13(z)−1=−36z1(1−z1z−1)(1−z1z) B23(z)=z3+z−3+110(z2+z−2)2304     +1087(z+z−1)+22122304.

Once the images have been processed with the cubic spline model the first derivative is applied both in *X*- and *Y*-axes direction obtaining new coefficients that are rescaled from 0 to 255 for visualization purposes. The results simulate raised areas in the image. This is due to the intensity changes produced on the image when converting from discrete to continuous coefficients with the previous transform.

A useful characteristic highlighted by the clinicians is that the *β*-spline transform keeps the original size of the calcium nodes.

Finally, the modulus of the *β*-spline filtering is calculated for exact detection. Areas with higher intensity values and greater variation of these values will have greater prominence. A breast lesion over an enhanced background is obtained with this technique. This method enhances both masses and microcalcifications.

(iii) *Adaptive Filtering*. It is based on the fact that the outline of objects within an image is characterized by discontinuities. It tries to find and mark such discontinuities by means of least squares, in a neighbourhood defined by a mask known as the 2D lattice structure. This algorithm is useful for microcalcification detection.

The lattice structure is composed of multi-inputs and multioutputs defined by a coefficient of variation. The outputs are backward and forward prediction errors, *e*_*M*_^*b*^(*n*) and *e*_*M*_^*f*^(*n*), calculated simultaneously. Thus, the algorithm is based on a 2D predictor-estimator within a region, *R*, of size (*m* + 1, *n* + 1). The output of a signal at point *x*(*n*_*i*_, *n*_*j*_), with *i*, *j* belonging to *R*, is the estimation of the following element, calculated as a linear combination (l.c.) of the (*m* + 1, *n* + 1) elements of the lattice structure; that is,

(7)  x−(n1,n2)∑i,j,Rai,jx(n1−i,n2−j)=xf(n1,n2)x−(n3−m,n4−n)∑i,j,Rai,jx(n3−i+1,n4−j+1)=xb(n3,n4).

Once the prediction error is obtained for each pixel, a threshold, *γ*, is defined to establish if the process is stationary or not. From a geometrical point of view, the prediction error is the projection of the input signal over the l.c. calculated with the elements of the 2D lattice structure. Thus, an angular coordinate, *θ*, is defined as the angle from the signal to the prediction space; those values of *θ* close to 0 mean a good approximation. The threshold, *γ*, is therefore defined by the following equation:

(8) $$\begin{array}{c} \gamma_{R}\left(n\right)=\cos^{2}\left(\theta \right) \end{array}$$

being close to 1 when the prediction error is small and varying with discontinuities.

As shown in (8), *γ* also varies with the prediction process; thus, the threshold must be based on the gradient image. The FP fraction detection is dependant on a good selection of the threshold. Finally, those pixels detected by the adaptative filter are grouped together by a region growing algorithm. In order to avoid FP the region growing algorithm discharges those ROI smaller than a certain size (of about 5 pixels).

The algorithm works well for fatty tissue and small lesions, being satisfactory to detect suspicious and isolated lesions. However, it may fail with many FP detections if a proper configuration of *γ* is not selected. The number of FP is higher on dense and fatty-glandular tissues due to their low contrast; it has been shown how the *β*-spline algorithm works better for this type of tissue.

## 5. Results and Discussion

**Figure 7:**
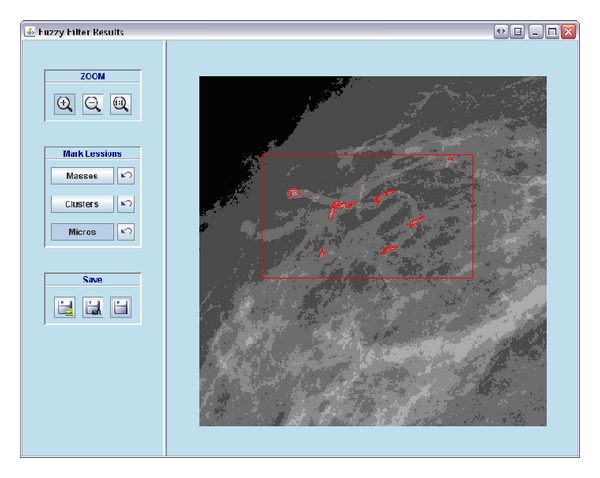
A marked cluster of microcalcifications.

An EHR and a CAD system have been developed and integrated. Figures 2 and 3 show the appearance of the mammographic EHR. It is possible to add, edit, and save data, test results, generate reports, and show images associated with a radiological study. These images are displayed on the CADe application where it is possible to use editing, preprocessing, and processing tools to find and measure lesions (see Figures 5 and 6). Moreover, the CAD system provides tools to mark lesions once they have been located (Figure 7). After marking lesions, results can be stored persistently through the database and the DICOM-SR output files.

### 5.1. Results of the CAD Lesion Detection Algorithms

**Figure 8:**
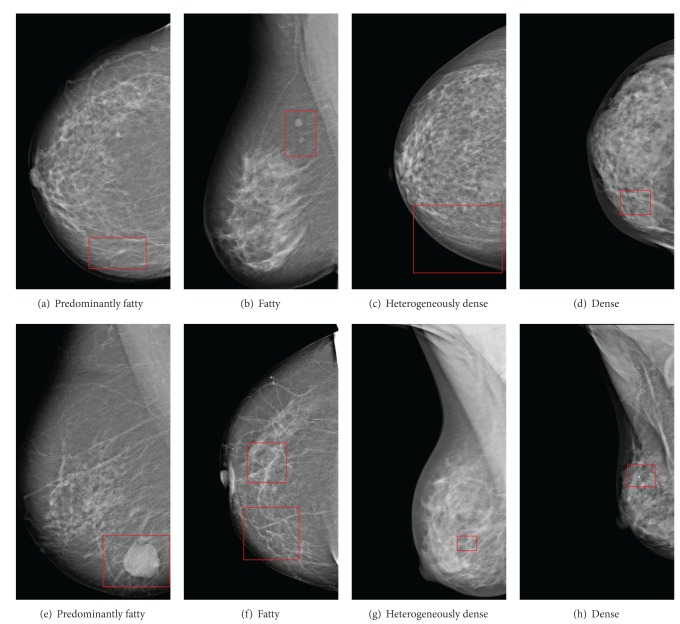
Some test images with (a) spiculated mass, (b) two well-defined masses, (c) region microcalcifications, (d) microcalcifications, (e) a well-defined mass, (f) a rounded microcalcification and some vascular microcalcifications, (g) a small microcalcification, and (h) a microcalcification.

**Figure 9:**
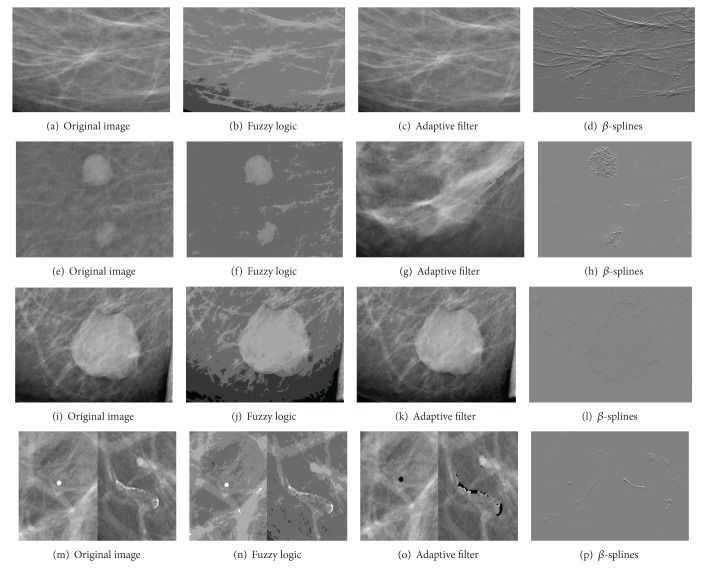
Results of the processing tools on predominately fatty (a, e) and fatty images (b, f) from Figure 8.

**Figure 10:**
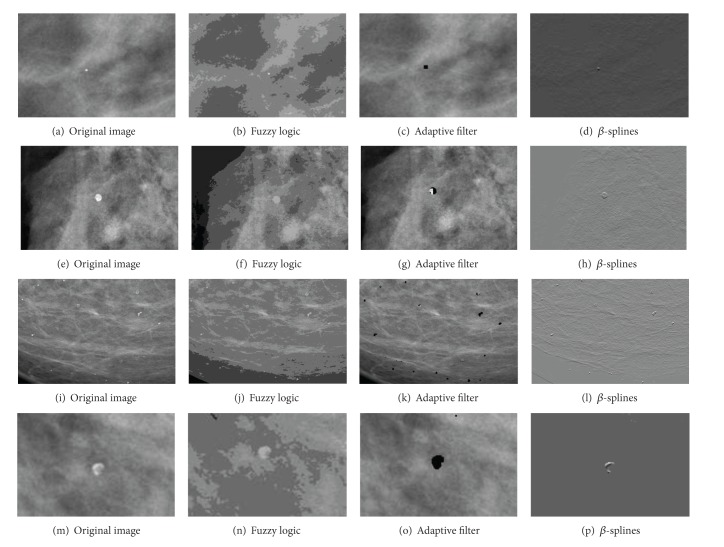
Results of the processing tools on heterogeneously dense (c, g) and dense images (d, h) from Figure 8.

The processing tools implemented into the CADe system have been tested and qualitatively validated by expert clinicians at Hospital General Universitario of Ciudad Real. The results obtained with the CAD segmentation algorithms on eight of the images are illustrated in Figures 8, 9, and 10. These images represent the four tissue types specified by the ACR in the BI-RADS Atlas [24], predominantly fatty, fatty, heterogeneously dense, and dense [24]. In addition, each image belongs to different patients and projections (CC and MLO).

Figure 8(a) shows a spiculated mass at the bottom of the breast tissue and no microcalcifications were present (Figures 9(a)–9(d)). It has a predominantly fatty breast tissue. Making the contrast enhancement using fuzzy clustering we can obtain an output that adequately defines the contours of the lesion. This new image provides uniformity to the background and highlights the lesions assigning similar values of neighbouring pixels. The result from the adaptive filter shows a small mark in the spiculated lesion. In case of using *β*-Spline it is possible to get an image that enhances the lesion clearly distinguished from the background.

In Figure 8(b) it is possible to see two well-defined masses on top of the mammographic tissue (Figures 9(e)–9(h)). The breast tissue in this case is fatty. As in the previous case this image does not have microcalcifications and making the contrast enhancement using fuzzy clustering an output that adequately defines the contours of the lesion is obtained. The adaptive filter marks some FP points in a dense area of the parenchyma. Using *β*-Splines it is possible to get an image that highlights the lesions through a raised area.

Figure 8(c) contains a heterogeneous tissue. There are microcalcifications at the bottom of the breast tissue in this case (Figures 9(i)–9(l)). With fuzzy logic clustering an image that highlights the microcalcifications and their contour is achieved. In this case, the adaptive filter does not produce marks. Using *β*-Splines the image obtained as output presents a raised effect in the lesions and makes these quickly and clearly visible.

Figure 8(d) contains an extremely dense mammographic tissue. At the bottom of the mammographic tissue appears an isolated microcalcification (Figures 9(m)–9(p)). With fuzzy logic clustering it is possible to obtain an image that facilitates lesion recognition and contour definition. The adaptive filter marks correctly the microcalcification but, in this tissue type, some false-positives appear caused by the high intensity of the breast tissue. By means of *β*-Splines an image with an homogeneous background and a small raised area in the lesion is obtained.

In Figure 8(e) it is possible to see a large well surrounded mass at the bottom of the breast tissue (Figures 10(a)–10(d)). The breast tissue does not present microcalcifications in this case and follows a predominantly fatty pattern. Enhancing contrast by using fuzzy clustering allows to define the contours of the lesion correctly. In case of using *β*-Spline present a raised area from the background. 

Figure 8(f) has an isolated microcalcification in the center of the image and some vascular microcalcifications at the bottom (Figures 10(e)–10(h)). The breast tissue in this case is fatty. With the contrast enhancement by using fuzzy clustering an output that adequately defines the contours of the lesions is obtained. The adaptive filter marks correctly the microcalcification in both cases. In case of using *β*-Spline, the image obtained presents a raised area distinguishing the lesion from the background.

Figure 8(g) contains a heterogeneously tissue. There is only one small microcalcification in the center of the breast tissue (Figures 10(i)–10(l)). With fuzzy logic clustering an image that highlights the microcalcifications and their contour is achieved. Applying the adaptive filter the microcalcification is detected and marked correctly. Using *β*-Splines, the image obtained as output presents a raised area that makes the lesion clearly visible.

Finally, Figure 8(h) contains an extremely dense mammographic tissue. This image contains also a microcalcification (Figures 10(m)–10(p)). With fuzzy logic clustering it is possible to obtain an image that defines correctly the contour. The adaptive filter marks correctly the microcalcification. By means of *β*-Splines an image with a homogeneous background and a raised area in the lesion is obtained.

The use of a specific algorithm or another depends on the lesion that we are looking for. For masses, the algorithm which provides better results is the fuzzy logic clustering method. This algorithm helps to define lesion contours and offers computational time significantly lower than the rest of the methods. In case of detecting microcalcifications we can arrive at different conclusions depending on the tissue type from the mammogram. On mammograms whose tissue type is extremely dense the algorithm that gives better results is the algorithm based on *β*-Spline contrast enhancement. With this tissue type, using the fuzzy logic clustering algorithm does not adequately distinguish microcalcifications. For the other tissues, both, the adaptive filter or the *β*-Spline based-algorithm, may be used.

The filtering presented has shown to be successful in highlighting breast lesions on different types of tissues. It is worth mentioning the comments made by the clinicians. The tools improve the resolution, in terms of the detectability of lesions, and additionally, they are able to distinguish their degrees of attenuation.

### 5.2. Effectiveness of the Whole System

In order to test the effectiveness of the implemented system a study composed of 28 cases belonging to 26 patients, that is, a total of 194 images from the parenchyma area, has been carried out. The 194 images are divided into 82 images without lesions, 89 with benign lesions, and 23 with malign lesions.

The study was carried out by 4 radiologists from local hospitals. The EHR was populated with general information of the patient and specific information which may influence breast cancer diagnosis as well as the image density and therefore the amount of false positive detections.  Table 1 shows the results of the complete system, comparing the performance of the CADe with and without using the specific information provided by the EHR. The algorithms incorporated into the CAD do not distinguish between benign and malign lesions. However, when the specific EHR information was used and this information was hinted at a malign lesion, then if the CAD detected a lesion this was highlighted in red, in other cases the adaptive filter was run again with different parameters. The adaptive filter was also run twice with different parameters in the case where a lesion was detected and the data from the EHR was not sensitive of being cancer.

The results were validated by the radiologists, comparing the automatic results given by the CAD with those given by the radiologist. Thus, the integration of the EHR information was useful to increase the number of true positive detections (TPDs) and reduce the number of false positives (FPDs). Moreover, the evaluation of the usability of the system made by the clinicians through some questionnaires, like in [46], found the whole system very useful because (a) it allows to do prospective studies and review previous detected lesions, (b) it has all the useful information integrated in a single application, and (c) the EHR may help classifying the lesions by means of rule based classification methods and developing dedicated ontologies.

### 5.3. Computational Time of the CAD Tools

**Figure 11:**
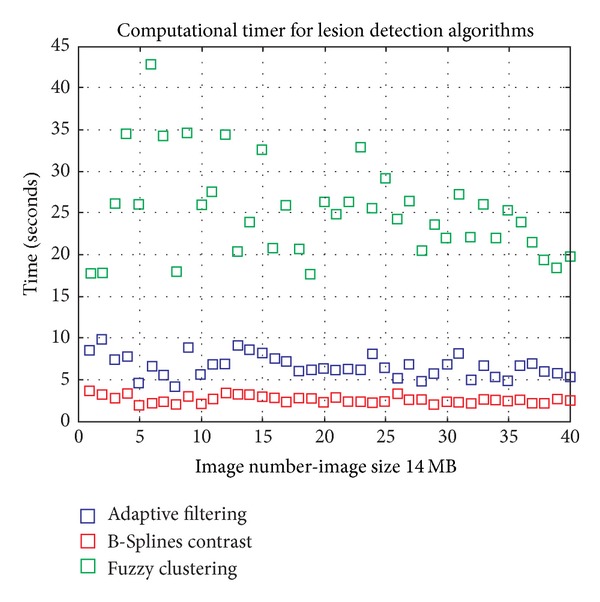
Computational times of image processing algorithms.

**Figure 12:**
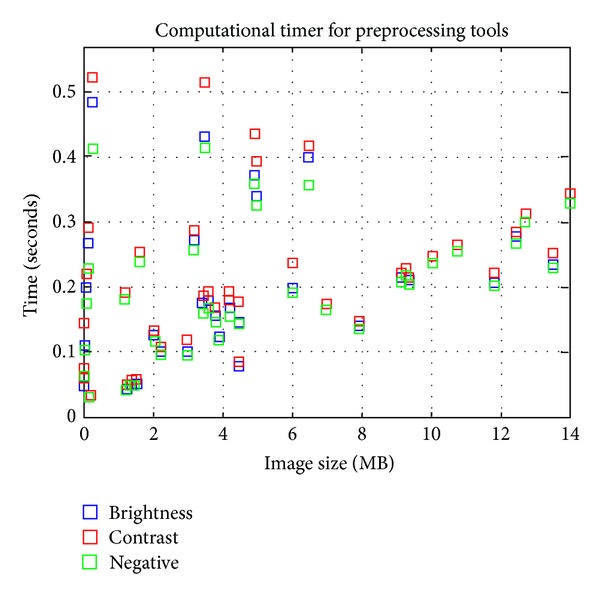
Computational times of preprocessing tools.

In any system it is important to take into account the computational time required to obtain the results. The computational times of the preprocessing and the image processing algorithms have been obtained during the system tests. All times were obtained with an Intel Xeon CPU E5440 2.83 GHz with 3 GB of RAM and with a precision of milliseconds.  Figure 11 shows the computational times for a dataset with a subset of 40 mammograms in DICOM format with 3328 × 4084 pixels.  Figure 12 shows the computational times of the preprocessing tools using different file formats.

 The results show computational times lower than 1 minute for all detection algorithms, being under 10 seconds for the adaptive filtering and *β*-Splines. Moreover, the computational time for the preprocessing tools is also very low, under 0.5 seconds. These computational times make it possible to integrate these tools in the EHR and their use in the daily work.

## 6. Conclusions

In some medical centres it is still common that medical tests results are printed on paper and the patient has the responsibility of carrying these results to the hospital. This caused delays and, sometimes, the results could be damaged. Moreover, in case of using radiological tests, an X-ray view box and a magnifying glass are necessary to view and amplify the results.

Thanks to the EHRs, as the one developed in this work, it is possible to access and process clinical stored data about patient information and clinical tests and then solve the above-mentioned problems.

Furthermore, the use of digital imaging in breast cancer detection makes it possible to develop tools to assist radiologists in the diagnosis of this disease. In this work we have implemented a CADe system that integrates some tools to offer adjustments of the image zoom, mechanisms to change image brightness and contrast, the ability to see more clearly some areas, and the possibility to take measurements with a few mouse clicks. Such tools are now, according to expert radiologists in breast cancer, widely used in their daily work.

We also include in the CAD system more complex algorithms to help radiologists detect the two most common types of mammographic tissue lesions that are sometimes associated with breast cancer.

The fact that the CAD application has been developed to be integrated within the EHR makes all the applications that a specialist need options within a main application and promotes the usability of the entire application. Moreover, using information from both the EHR and the CADe system helps to provide a more complete diagnostic and therefore to reduce the number of FP detections and increase the TP.

Finally, using the DICOM standard to develop this application (DICOM for image management and storage and DICOM-SR for reports) facilitates the compatibility with other programs. The use of DICOM-SR for reports is also a novelty of the CADe implemented in this work.

Further ongoing work is devoted to provide some tools to classify the type of lesions into benign and malign, using also the specific information of the EHR.

## Figures and Tables

**Table 1:** Performance of the whole system (% TPD and % FPD).

% Detections	CADe	CADe + EHR
TPD	75%	85%
FPD	25%	12%
